# Telerehabilitation in Older Thai Community-Dwelling Adults

**DOI:** 10.3390/life12122029

**Published:** 2022-12-05

**Authors:** Chernkhuan Stonsaovapak, Viboon Sangveraphunsiri, Weerachai Jitpugdee, Krisna Piravej

**Affiliations:** 1 Department of Rehabilitation Medicine, King Chulalongkorn Memorial Hospital, Bangkok 10330, Thailand; 2 Department of Rehabilitation Medicine, Faculty of Medicine, Chulalongkorn University, Bangkok 10330, Thailand; 3 International School of Engineering Faculty of Engineering, Chulalongkorn University, Bangkok 10330, Thailand

**Keywords:** community, fall, healthy elderly, physical performance, rehabilitation, telemedicine, wearable devices

## Abstract

**Simple Summary:**

In this work, we created a new telecommunication technology for evaluating and giving consultation or treatment from a remote area. By employing wearable sensor technologies on healthy older participants, we hoped to demonstrate the benefits of teleconferencing systems for assessing physical performance, walking ability, and fall risk and offering exercise regimens. The initiative was carried out in four rural areas in Thailand. The group tele-exercise program included 123 participants in total and lasted for 8 weeks. We found that tele-exercise enhances physical performance and lowers the risk of falls in healthy older adults living in a community compared to an initial examination. The participants’ total satisfaction score was remarkable. We hope that these results could lead to building a full component of the telerehabilitation system in Thailand and increase accessibility for patients in rural areas.

**Abstract:**

To investigate the impact on physical performance and walking abilities associated with fall risk and disability in the senior population, we created a telerehabilitation system. This is a multi-site, community setting, pre–post experimental study. We recruited participants from four rural areas in Thailand. All participants received eight weeks of tele-exercise, three sessions per week, via the telerehabilitation system. After the intervention, all participants underwent the Short Physical Performance Battery (SPPB), Timed Up and Go (TUG) test, and the six-minute walk test (6MWT) using a wearable sensor system. A total of 123 participants participated in the study and 2 participants dropped out while conducting the study, thus 121 participants were included in the analysis. In comparison to the baseline, we discovered a considerable improvement in the SPPB score (0.65 ± 0.22, *p* < 0.001), TUG (−1.70 ± 0.86, *p* < 0.001), and 6MWT (10.23 ± 7.33, *p* = 0.007). Our study demonstrates the benefits of telerehabilitation on SPPB, TUG, and 6MWT related to disabilities and fall risk. This telerehabilitation technology demonstrated its viability in the community environment and demonstrated its capacity to offer fundamental components of remote rehabilitation services within the healthcare system.

## 1. Introduction

Bioengineering technology is now developing rapidly, resulting in several innovative healthcare platforms and products [1,2]. Telerehabilitation is a recent development that saw increased use during the COVID-19 pandemic since it allows for the delivery of rehabilitation services to patients’ homes while also preventing the spread of disease. Increased patient accessibility to evaluation, assessment, monitoring, intervention, coaching, education, and consultation from healthcare professionals is the goal of telerehabilitation. Telemedicine offers a variety of direct and indirect benefits, including fewer traveling costs and less time, increased access to healthcare in rural or isolated locations, improved patient–physician relationships throughout treatment, and the promotion of general health issues [3]. However, to measure joint range of motion, perform functional tests, or even analyze gait, a precise and dependable instrument must be able to detect movement from a distant place [4]. Optical motion identification, 3D depth camera-based movement detection, robot-based solutions, and wearable sensor-based systems are just a few of the alternatives to employing standard goniometers to assess body movement [5].

During the COVID-19 pandemic, numerous emerging technologies were applied to provide rehabilitation or healthcare services in various areas of diseases in the aging population, such as musculoskeletal problems, stroke, and traumatic brain injury, from remote areas due to limited close contact [1,6]. Recent systematic reviews and clinical trials indicated the effectiveness of telerehabilitation in promoting physical function and therapeutic purposes in the elderly [7,8,9,10]. Saito et al. reviewed studies investigating the effectiveness of home-based telerehabilitation in elderly people living in Southeast Asia and reported equal or better effects on physical function compared to traditional rehabilitation [7]. The study by Velayati et al. showed comparable results of telerehabilitation and conventional rehabilitation in elderly people with various health conditions [8]. The use of telerehabilitation before and after arthroplasty of the knee and hip due to osteoarthritis or fracture, which are common problems in aging, resulted in positive effects on functional recovery after surgery [11,12,13].

Researchers focused on the significance of fall prevention strategies for the senior population, and there are growing replicated pieces of evidence supporting the benefit of using telerehabilitation in community-based settings. In a prior randomized clinical research study, the use of web conferencing technology for home-based group exercise and fall prevention programs was examined. The findings revealed that it might increase participants’ lower-limb muscular strength and endurance, as well as their sense of balance [14]. In another clinical trial investigating the impact of group resistance tele-exercise using developed video conferencing software, the intervention group outperformed the control group in terms of chair sit-and-reach scores, appendicular lean soft tissue, and total-body and lower-limb skeletal muscle mass [9]. A telerehabilitation system was employed in both studies to deliver interventions but not assessments.

We developed a system for telerehabilitation aimed to provide an assessment, as well as training, from any place and at any time. The system consists of a robotic machine for telecommunication that can detect real-time movement by using artificial intelligence (AI) algorithm processing on the 3D camera with a high-performance visual data processing system. With objective measurements, the therapist may assess if patients are performing the exercise correctly in real time. However, real-time feedback was not used in this investigation since the detecting sensor’s capability was exceeded by the number of participants in the group exercise. We created a wireless, wearable, 3D motion sensor that is fastened to the client’s body for assessment. For motion detection, the sensors include a high-resolution stereo vision camera, accelerometer, gyroscope, magnetometer, and barometer. Truncal deviation, gait speed, and functional testing might be evaluated using a high-performance graphic processing system portable CPU, a battery set, and a wireless data processing system. The evaluation results were stored in the cloud server via the internet; thus, the healthcare provider could analyze them later offline. The whole components of the telerehabilitation system are the benefits of this device. Instead of the 3D camera-based movement detection technology used in gait laboratory analysis, a wireless wearable motion sensor was used.

The primary objective of this study was to investigate the impact of the tele-exercise intervention on physical performance. Secondary objectives were to investigate the effects on walking abilities and physical performance associated with fall risk in the older adult population in the community setting. For these aims, we proposed the deployment of a telerehabilitation system consisting of a robotic machine for telecommunication during group exercise without real-time feedback and a wireless, wearable motion sensor for the evaluation part. In addition to improving the ability of hospitals in rural areas to offer fundamental components of remote rehabilitation services in the healthcare system, we expect that the results can be utilized as a model for setting up the community’s telerehabilitation system.

## 2. Materials and Methods

This study was a multi-site, community-based, pre–post experimental study conducted under the permission of the Institutional Review Board of the Faculty of Medicine, Chulalongkorn University, Bangkok, Thailand (IRB no.603/64) and was registered in the Clinical Trials Registry (TCTR20210830003 Released date 30 August 2021).

### 2.1. Participants

Participants were drawn from four rural communities in Thailand: Lampang, Chaiyaphum, Chumphon provinces, and Sichang island. With the cooperation of public health officers in each study site, they publicized our project to recruit participants. In this investigation, a convenience sampling method was employed.

According to the institutional review board, each participant received information and signed a consent form. The study comprised individuals older than 60 years old with a stable cardiovascular condition and who had given well-communicated and informed permission. Excluded from the program were seniors who used a walking aid or had a severe sensory or perception deficit, unstable medical conditions, musculoskeletal problems that caused difficulty in movement, or who were attending other exercise programs.

### 2.2. Wearable Sensor-Based System and Telerehabilitation System

**Figure 1:**
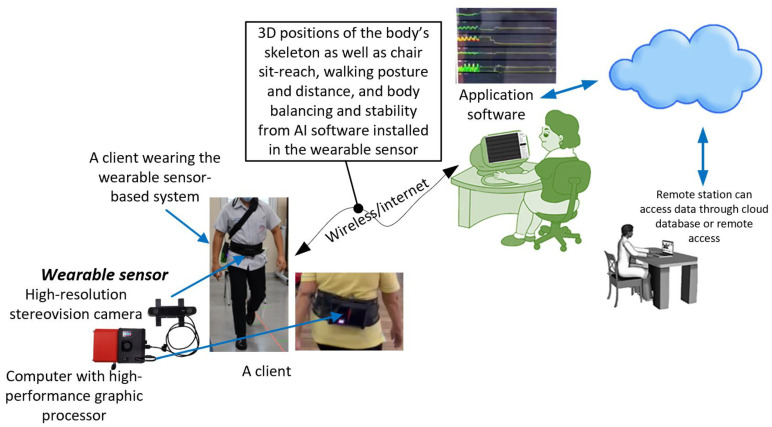
Wearable sensor-based system configuration.

While using the same basic hardware, the wearable sensor-based system is configured differently. A 3D camera and powerful visual data processing technology make up the wearable sensor-based system. An AI algorithm is used to estimate the 3D positions of the body’s skeleton, including the nose, neck, right eye, left eye, right ear, left ear, right shoulder, right elbow, right wrist, left shoulder, left elbow, left wrist, right hip, right knee, right ankle, left hip, left knee, left ankle, as well as chair sit–reach, walking posture and distance, and body balancing and stability. Figure 1 shows the configuration of the wearable sensor-based system. The client is outfitted with the sensors and graphics processing as seen. The e-application software installed at a station computer receives the data wirelessly and process them. The processed data can be kept on a cloud system and are available for online data retrieval.

**Figure 2:**
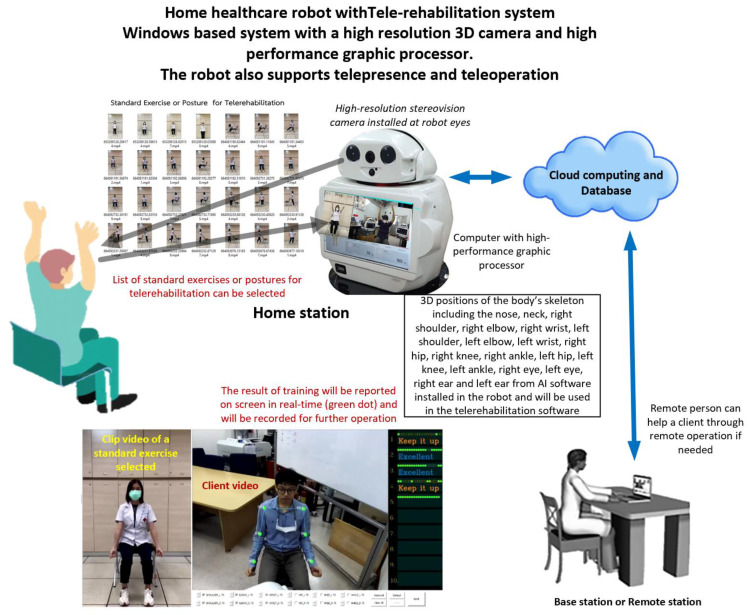
Telerehabilitation system.

The wearable sensor-based system is similar to the configuration of the telerehabilitation system, which comprises a 3D camera with a high-performance visual data processing system. The 3D camera is implanted in the eyes of the home healthcare robot. The motion data captured are processed by AI software to report in real time the performance of the rehabilitation. Through the remote operation feature of the home healthcare robot, a remote operator may occasionally be able to assist the customer in setting up the rehabilitation program (Figure 2).

### 2.3. Intervention

At each study location, participants attended a group tele-exercise. To increase their lower-limb strength, endurance, and balance, they adhered to a video clip of aerobic dance for the older adults under medical supervision for 30 min, three times per week, for eight weeks. Physiotherapists monitored the workout quality using the telerehabilitation system.

The exercise program followed the American College of Sports and Medicine guideline that addressed the importance of aerobic, strength, flexibility, and neuromotor training in older adults [15]. The first part of the training program started with a five-minute warm-up consisting of shoulder circles, forward lunges, side lunges, mini-squats, and ankle circles. The main part of the program was an exercise in the form of aerobic dancing focused on the dynamic movement of the lower extremities, weight-bearing calisthenics, and stepping in multidirectional movements to improve dynamic balance, which lasted 30 min. The last part was a cool-down period, in which participants performed stretching exercises of the shoulder, triceps, anterior chest wall, anterior neck, trunk, hip flexor, hamstring, and calf muscles.

The supervised therapists instructed all participants to use the talk test to measure the intensity of exercise. We prescribed moderate-intensity aerobic exercise, meaning participants could talk but could not sing during the training period [16]. A recent systematic review of exercise intervention improving physical performance in the elderly reported that intervention times ranged from 4 to 52 weeks [17]. We chose the eight-week exercise program due to the practicality of conducting the multi-site project.

### 2.4. Outcome Measurements

For baseline information, we logged gender, age, weight, height, body mass index (BMI), educational level, comorbidities, and frequency of falls in the previous year. Physical performance assessed by a Short Physical Performance Battery (SPPB) was the main result. The test examined the utility of lower-limb function for fall risk assessment in the elderly population [18]. Low SPPB scores have been linked to falls, frailty, sarcopenia, mobility-related disability, daily activity living disability, and increased all-cause mortality [18,19,20,21]. The SPPB test consists of a balance test (side-by-side stand test, semi-tandem stand, tandem stand), a four-meter gait speed test, and a chair–stand test. It was simple to use, only took a small amount of time to assess, was safe for senior people, and had great validity and reliability [22]. The SPPB score can be stratified into four classes: 0–3 very low performance, 4–6 low performance, 7–9 intermediate performance, and 10–12 high performance [23]. The Timed Up and Go (TUG) test and the six-minute walk test (6MWT) provided secondary outcomes. Both assessments may be able to forecast the likelihood that community-dwelling seniors may fall [24,25]. Using the self-reported patient questionnaire that was translated into the Thai language with permission from a prior study on telemedicine in Parkinson’s disease, we also assessed participant satisfaction [26]. It consists of 10 items with a five-point Likert scale: 3 items for the technical aspect and 7 items for quality and privacy issues of the telerehabilitation system.

### 2.5. Sample Size Calculation

Sample size calculation was based on previous studies using a two-dependent group formula. The minimal clinically important difference in the SPPB was a 1.0 score [27], standard deviations were 1.32 and 2.99 [28] with a power of 90% and α of 0.05, and a total of at least 112 participants had to be enrolled. Power analysis and determination of the sample size were done prior to the study.

### 2.6. Statistical Analysis

For data analysis, STATA version 15.1 was employed. With the help of a histogram and skewness, normality was evaluated. While categorical data were given as total number and percentage, continuous data were represented as mean and SD. To compare the outcomes before and after the intervention, a paired *t*-test was performed. We compared the physical performance classification and fall risk of participants at baseline and post-intervention using the McNemar test. The statistical significance level was set at a *p*-value of <0.05.

## 3. Results

### 3.1. Participants

**Scheme 1:**
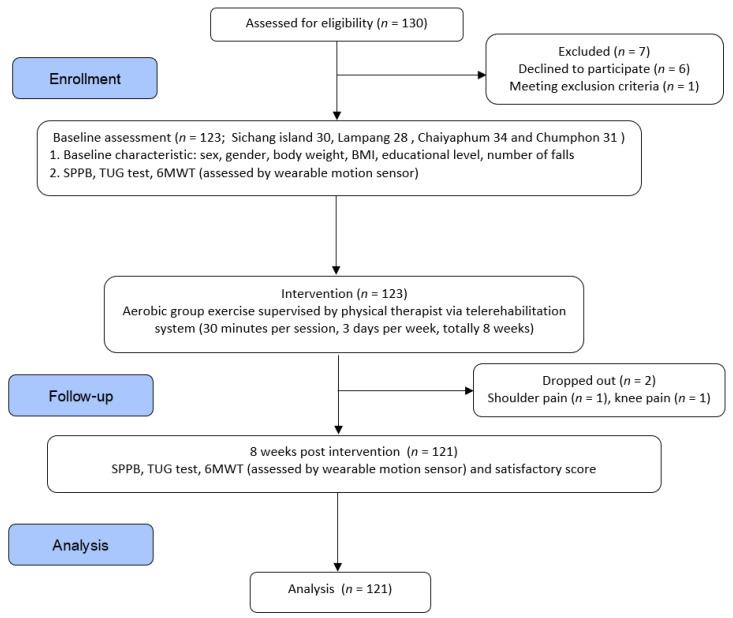
Study flow diagram.

The participants were recruited from December 2021 to May 2022. Overall, 130 participants were enrolled in the study. One individual who met the exclusion criteria was one of seven who were not able to participate in the group activity. In total, 123 people were eligible for the study: 30 from Sichang island, 28 from Lampang province, 34 from Chaiyaphum, and 31 from Chumphon were eligible for the study. Due to one’s shoulder pain and the other’s knee pain, two study participants withdrew from the experiment. After the intervention, 121 participants in total were included in the data analysis (Scheme 1). Following the intuitive board review procedure, all participants received information and signed consent forms. The demographic data are presented in Table 1.

### 3.2. Physical Performance and Walking Ability Outcomes

The participants’ scores of the SPPB (0.65 ± 0.22, *p* < 0.001), TUG (−1.70 ± 0.86, *p* < 0.001), and 6MWT (10.23 ± 7.33, *p* = 0.007) eight weeks following group exercise through the telerehabilitation system had significantly improved compared to the baseline score (Table 2).

The majority of the participants exhibited moderate to high physical performance at baseline when the SPPB scores were divided into the four performance groups of very low, low, intermediate, and high performance. No participants were classified as very low performance, and only 10% of participants were stratified in the low performance category. The earlier study [29] that investigated the predictive efficacy of SPPB to fall risk used a cut-off point of 10.5 as it determined that 68.6 percent of participants had an increased fall risk at baseline assessment. In comparison to the baseline evaluation, we found a significant improvement in physical performance classification (*p* = 0.003) and a decrease in the risk of falling (*p* < 0.001) compared to the baseline assessment after the intervention (Table 3).

### 3.3. Satisfaction Outcome

Overall, 95 percent of participants expressed that they would continue to use the telerehabilitation system after the study was finished, and participants across all study locations reported an overall satisfaction score for the system of 47.08 out of 50. The survey response rate was 97.6% (Table 4).

## 4. Discussion

The goal of this study was to develop a system for telerehabilitation that would enable real-time assessments, monitoring, and interactions between physiotherapists and older adults in rural areas and to investigate its effects on the improvement of the SPPB score, TUG test, and 6MWT and level of satisfaction among the older Thai community-dwelling adults. In comparison to the starting point, all primary and secondary outcome scores were improved significantly. In addition, we discovered that this intervention reduced the chance of falling and improved the classification of physical performance when compared to the baseline assessment. Participant satisfaction was remarkably high overall. The remote assessment and intervention increased accessibility, reduced cost, and allowed healthcare providers to follow up or monitor during the intervention. Additionally, our study demonstrated the viability of a telerehabilitation system in community settings.

As people age, their risk of falling increases due to impaired muscle function, poor balance, and unsteady gait [30]. Physical performance can be improved by working on balance, muscle strength, and endurance [31,32]. The prognostic capability of the SPPB test to predict fall risk, disability, and mortality has previously been extensively studied [18,19,20,21,23]. After the intervention, we saw a considerable improvement in the SPPB score, even though the change score of 0.65 only showed a marginally significant change [27]. Substantial changes in the SPPB score are considered to be 1.0 points [27]. This may be explained by the fact that the majority of the older adults who participated in our study were initially categorized as having high physical performance levels, which may have contributed to the test’s ceiling effect. Nevertheless, the results reveal that, after receiving the intervention, participants who were categorized as being in the low physical performance group [23] and at high risk of falling [29] had improved performance and reduced fall risk.

Although they do not appear to be clinically significant, with 2.08 s for the TUG test and 50 m for the 6-min walk test, the results of the secondary outcomes revealed statistically significant improvement [27,33]. The prediction value of fall risk was thoroughly investigated using the TUG test. When used alone, the TUG test showed a limited ability to predict fall risk in senior people living in the community, according to meta-analyses, which suggested utilizing a mix of functional tests already present in the SPPB test [24,34]. The 6MWT, meanwhile, may indicate a person’s capacity for physical activity [35]. The exercise intervention time in previous studies was in a wide range of 4 to 52 weeks [17]; however, a longer duration of exercise could induce more improvement in physical fitness according to the dose–response relationship [36].

Our findings are consistent with a recent systematic review that found substantial evidence for the significance of exercise in enhancing physical fitness in the elderly, particularly aerobic exercise mixed with strengthening activities [17]. Alpozgen et al. investigated the effects of tele-exercise consisting of strengthening, balance, and flexibility exercise to maintain physical fitness, quality of life, and mood in older people by conducting a randomized controlled trial. The results showed that exercise through a real-time video conference system could improve mood and physical fitness, evaluated by the Senior Fitness Test Battery, and maintain quality of life [10]. Wu et al. conducted a three-arm randomized controlled trial, using telecommunication-based exercise through the videoconferencing system, community-based exercise, and home video-based exercise to explore the compliance and effectiveness of a Tai Chi exercise program in elderly people at risk of falls. They found that telerehabilitation and community-based exercise demonstrated better compliance and fewer falls during the study period [37]. However, the results of the TUG test did not show significant changes within and between groups, which could be due to the specificity of the type of exercise that contradicts the results of our study. A previous randomized controlled trial investigated the effects of strengthening exercise through the videoconferencing system on sarcopenia-related factors, as well as functional fitness, in older people [9]. There were significantly better improvements in muscle mass and chair sit-and-reach length reflected in participants’ fitness in a tele-exercise group. The effects of tele-exercise in our study also improved the SPPB score, which assessed lower-limb functions related to sarcopenia [20]. Importantly, we found that the telerehabilitation system was feasible and acceptable to use in older adults, which corresponded to the results of the previous systematic review reporting the usability of telemedicine in various health outcomes [6]. The effectiveness and viability of a telerehabilitation system in community settings were also supported by this investigation, which builds on earlier clinical trials [9,14,37].

The utilization of a wireless wearable sensor-based system for an assessment, along with a telerehabilitation system for communication and intervention, is the study’s key strength. We were able to assess participants’ trunk mobility from a remote location using a 3D camera with a high-performance visual data processing system, an AI algorithm that estimated body locations, and a portable battery system. Functional testing, gait speed, and distance were recorded and saved in the cloud system. The gadgets were employed in place of the 3D gait laboratory analysis, which cut down on testing time and the cost of the participants’ journeys to our hospital, and also served as a working prototype of a complete element of remote rehabilitation services in Thailand.

Our research had some limitations. First off, since this was the first time we used our ground-breaking telerehabilitation in a multi-site community environment, it was a single-arm clinical trial. We tested the system’s viability in actual practice. However, the outcomes could be impacted by uncontrolled factors other than the effect of the intervention. The randomized controlled trial study design would instead achieve the aim of com-paring the effectiveness of the telerehabilitation system to conventional rehabilitation. Second, there were several tools for fall risk assessment, but we used a fall risk classification based on a previous study [29], which was classified by the SPPB score, which might not comprehensively reflect the fall risk of participants. Third, because the majority of our individuals had high and intermediate physical performance, we were unable to stratify to subgroup analysis. Lastly, the home healthcare robot in the telerehabilitation system, which could report real-time feedback to therapists and set up the rehab program for clients, was also not used. Our research is intended to serve as a template for future home-based telerehabilitation programs in Thailand.

## 5. Conclusions

Our findings indicate that, after participating in group tele-exercise, there were favorable impacts on the SPPB, TUG test, and 6MWT scores in healthy older adults. Additionally, our study demonstrated the system’s viability when applied in a community-based setting, and participants expressed appreciation for the telerehabilitation system.

## Figures and Tables

**Table 1:** Demographic data for the participants.

Variables	Female (*n* = 103)	Male (*n* = 18)	Total (*n* = 121)
Age (years), mean (SD)	67.0 (5.6)	69.7 (5.0)	67.4 (5.6)
Weight (kilograms), mean (SD)	60.9 (9.4)	66.4 (14.1)	61.7 (10.3)
Height (centimeters), mean (SD)	154.8 (5.5)	164.9 (8.3)	156.3 (7)
Body mass index (BMI), mean (SD)	25.4 (3.6)	24.4 (4.8)	25.2 (3.8)
Study site, *n* (%)			
Sichang island	25 (24.3)	3 (16.7)	28 (23.14)
Lampang	23 (22.3)	5 (27.8)	28 (23.14)
Chaiyaphum	30 (29.1)	4 (22.2)	34 (28.11)
Chumphon	25 (24.3)	6 (33.3)	31 (25.61)
Education, *n* (%)			
Elementary	55 (53.4)	10 (55.5)	65 (53.7)
Secondary	31 (30.1)	5 (27.8)	36 (29.8)
Higher education	17 (16.5)	3 (16.7)	20 (16.5)
Frequency of falls in the past year, *n* (%)			
0	81 (78.6)	17 (94.4)	98 (81.0)
1–2	18 (17.5)	1 (5.6)	19 (15.7)
>2	4 (3.9)	0 (0)	4 (3.3)
Comorbidities, *n* (%)			
Hypertension	47 (45.6)	9 (50.0)	56 (46.3)
Diabetes mellitus	16 (15.5)	5 (27.8)	21 (17.4)
Dyslipidemia	51 (49.5)	10 (55.6)	61 (50.4)
Heart disease	5 (4.9)	1 (5.6)	6 (5.0)
Back pain	8 (7.8)	1 (5.6)	9 (7.4)
Knee osteoarthritis	11 (10.7)	0 (0)	11 (9.1)

**Table 2:** Summary of clinical tests at baseline and 8 weeks post-intervention.

Tests	Baseline (*n* = 121) Mean (SD)	At 8 Weeks (*n* = 121) Mean (SD)	Pre–Post Mean [95% CI]	*p*-Value *
SPPB	9.18 (1.97)	9.83 (1.86)	0.65 [0.44, 0.87]	<0.001
TUG	15.10 (5.79)	13.40 (4.03)	−1.70 [−2.56, −0.85]	<0.001
6MWT				
Distance (meters)	333.58 (81.06)	343.82 (78.36)	10.23 [2.91, 17.56]	0.007
Speed (meter/second)	0.93 (0.23)	0.96 (0.22)	0.03 [0.01, 0.05]	0.007

* A paired *t*-test was used for the pre–post comparison. SPPB: Short Physical Performance Battery; TUG: Timed Up and Go; 6MWT: Six-minute walk test.

**Table 3:** Physical performance and fall risk categories differentiated by SPPB score at baseline and 8 weeks after intervention.

Classification	Baseline (*n* = 121)	At 8 Weeks (*n* = 121)	*p*-Value ^†^
Physical performance, *n* (%)			0.003
High	57 (47.11)	67 (55.37)	
Intermediate	54 (44.63)	52 (42.98)	
Low	10 (8.26)	2 (1.65)	
Very low	0 (0)	0 (0)	
Fall risk classification *, *n* (%)			<0.001
No risk of fall	38 (31.4)	52 (43.0)	
Increased risk of fall	83 (68.6)	69 (57.0)	

* Based on a cut-off value according to the study of Chen et al. [29], who classified an SPPB of <10.5 to be an increased risk of falling. ^†^ The McNemar test was used for the pre–post comparison.

**Table 4:** Satisfaction of participants with the telerehabilitation system.

Satisfaction	Study Site				
	Sichang Island (*n* = 28)	Lampang (*n* = 28)	Chaiyaphum (*n* = 34)	Chumphon (*n* = 30)	Overall (*n* = 120)
An overall score (total score of 50)	46.50	46.74	47.83	47.00	47.08
I feel that my privacy was respected.	4.82	4.61	4.97	4.60	4.76
2. I feel that the telerehabilitation system can give me a consultation on health issues and communicate with a doctor or physical therapist understandably.	4.78	4.54	4.38	4.60	4.57
3. I feel effectively rehabilitated through the telerehabilitation system.	4.71	4.68	4.71	4.67	4.69
4. I feel comfortable discussing and addressing all my concerns about health issues.	4.71	4.64	4.82	4.70	4.73
5. I feel relaxed while interacting with my physician and therapist.	4.75	4.71	4.76	4.70	4.73
6. I feel that I received benefits from using the telerehabilitation system.	4.86	4.71	4.97	4.73	4.83
7. There is no significant lag between sound and video during exercise.	4.18	4.64	4.94	4.73	4.64
8. I can hear my doctor.	4.54	4.71	4.79	4.73	4.70
9. I can see my doctor.	4.36	4.64	4.64	4.73	4.60
10. The telerehabilitation system saves my time and travels expenses.	4.79	4.86	4.85	4.80	4.83
Participants are interested in continuing to use the telerehabilitation system after the end of the project. *n* (%)	25 (89.29)	27 (96.43)	32 (94.12)	30 (100)	114 (95)

## Data Availability

The data presented in this study are available on request from the corresponding author. The data are not publicly available due to ethical issues.
